# Mandibular Talon Cusp in Primary Lateral Incisor: A Rare Case Report

**DOI:** 10.1155/2012/670745

**Published:** 2012-12-25

**Authors:** Swaminathan Kavitha, Haridoss Selvakumar, Rajendran Barathan

**Affiliations:** ^1^Department of Pedodontics & Preventive Dentistry, Faculty of Dental Sciences, Sri Ramachandra University, Chennai 600116, India; ^2^Department of Pedodontics & Preventive Dentistry, SRM Dental College, SRM University, Chennai 600089, India; ^3^Department of Pedodontics, Ultra Best Dental Science College, Madurai, India

## Abstract

A talon cusp is a dental anomaly commonly occurring in the permanent dentition compared to the primary dentition. It commonly affects the maxillary anterior teeth. In primary dentition, the most commonly affected tooth is the maxillary central incisors. This is a rare case report of a 5-year-old male patient with a talon cusp affecting the mandibular primary lateral incisor. Recognition and treatment of this anomaly at early stages is important to avoid complications.

## 1. Introduction

The term talon cusp defines a wide variety of accessory cusp-like structures, ranging from an enlarged cingulum to a well delineated anomalous cusp [1]. Mitchell first described this anomaly as a “process of horn-like shape, curving from the base downwards to the cutting edge” on the lingual surface of a maxillary central incisor of a female patient [2]. Mellor and Ripa named the accessory cusp talon cusp because of its resemblance in shape to an eagle's talon [3]. Radiographically a talon cusp is similar to normal tooth material, presenting with radioopaque enamel and dentin with or without an extension of the pulp tissue [4]. The diverse clinical manifestations of the anomaly have led a talon cusp to be described in many different ways, for example, supernumerary cusp, horn, hyperplastic cingulum, evaginated, odotome, cusped cingulum, accessory cusp, dens evaginatus and supernumerary lingual tubercle. Because there is a lack of strict diagnostic criteria with which to define a talon cusp, Mader suggested that the term should be reserved to describe only those anomalous cusps of succedaneous incisor teeth that project prominently from the lingual surface of the tooth, are morphologically well-delineated, and extend at least half the distance from cementoenamel junction to the incisal edge. Lesser-cusp-like formations in the cingulum area of succedaneous incisor teeth should be referred to as “enlarged cingula” or “prominent cingula” [5]. The anomalous talon cusp is composed of normal enamel and dentin with a varying extension of pulp tissue. It occurs more frequently in the permanent dentition than in primary dentition and shows a predilection for maxilla over the mandible [6]. With regard to tooth affinity, only central incisors are involved in the primary dentition and the maxillary lateral incisor is most often affected in the permanent dentition (67%), followed by the central incisors (24%) and canines (9%) [7]. Previously, the anomaly was considered to be rare, but current use of radiographs has disclosed that a talon cusp is not as rare as formerly thought [8]. Talon cusp may cause functional and esthetic problems; hence, early identification and timely management of the condition are very essential. A review of the literature reveals that the talon cusp has a striking predilection for the maxilla and that occurrence of talon cusp on mandibular incisors and supernumerary teeth is extremely rare [1]. The present case reports the occurrence of talon cusp on the lingual surface of primary mandibular lateral incisors which is a very rare finding.

## 2. Case Report

**Figure 1:**
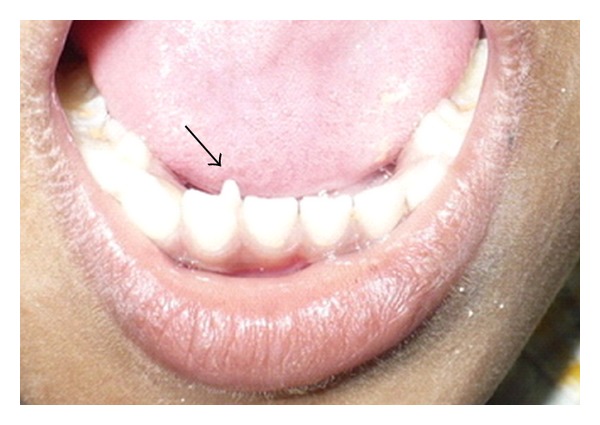
Talon cusp extending up to the incisal edge in 82.

**Figure 2:**
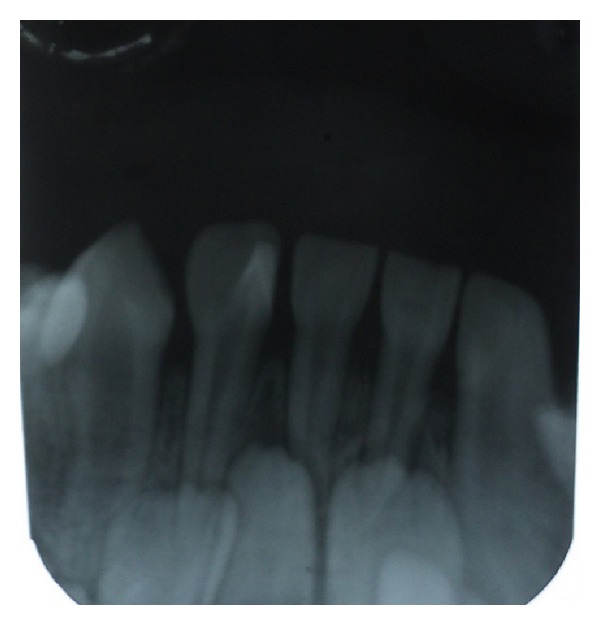
Radiographic image showing pulp extension into the talon cusp.

A 5-year-old male patient reported with the chief complaint of discolored upper front teeth. There was no relevant medical or dental history. On intraoral examination, dental caries was present in 51, 52, 61, 62, 74, 75, 84, and 85. An accessory cusp like projection was seen in relation to the lingual surface of 82 which extended from the cervical region to almost two-thirds of the incisal edge suggestive of a talon cusp (Figure 1). Intraoral periapical radiograph was taken in relation to 82 which not only confirmed the presence of a talon cusp in 82 but also revealed the presence of permanent tooth buds of 31, 32, 41, and 42 with no abnormal finding (Figure 2). The patient had no clinical problems associated with the presence of the talon cusp. Hence, no treatment such as grinding or reduction of the talon cusp was done. Restoration of the carious teeth was done and topical fluoride was applied. The patient was asked to report after every three months for review. 

## 3. Discussion

Davis and Brook defined the talon cusp as an additional cusp that predominantly projects from the lingual surface of primary or permanent anterior teeth, is morphologically well-delineated, and extends at least half the distance from cementoenamel junction to the incisal edge [9]. While the exact etiology of this anomaly is unknown [10], it has been suggested that a talon cusp might occur as a result/of an outward folding of the inner enamel epithelial cells and a transient focal hyperplasia of the mesenchymal dental papilla [11]. It has also been proposed that the talon cusp results from failed separation of a group of hyperactive cells that proliferate from the primordial cell. The hyperactivity of the primordial cells is genetically determined, but the degree of hyperactivity is influenced by environmental factors [12]. It has been suggested that improper action between ectoderm and mesoderm during the odontogenesis process on epithelial bulging may be the etiological factors for the formation of a talon cusp [13]. Family histories of cases reported previously revealed that sometimes the talon cusp affected patients who had consanguineous parents [1]. However, sporadic occurrences of this abnormality probably are induced by trauma or other localized insults affecting the tooth germ [14]. In 1999, the first and only case of a talon cusp on a primary mandibular incisor in an Indian girl with a talon cusp on the primary mandibular left lateral incisor was reported which extended to the middle third of the crown [10]. In the present case report, a talon cusp is seen in the lingual surface of a primary mandibular right lateral incisor which is not a very common finding. There are not many cases reported in the literature with a talon cusp in mandibular teeth. Radiographically, the talon cusp is visible as a radioopaque structure [10]. Most authors reported the talon cusp to be composed of normal enamel, dentin, and pulp [5]. In Mader and Kellogg's view, large talon cusps, especially when separated from the lingual surface of the tooth, seem more likely to contain pulpal tissue [15]. The radiograph in this case report indicated possible presence of pulp in the talon cusp. Lee et al. demonstrated that individuals with talon cusps on a deciduous maxillary lateral incisor showed a high proportion of odontogenic abnormalities in the permanent successor [12]. However, in our case there was no abnormal finding seen in the permanent tooth buds. Clinical findings along with the patient's presenting complaints will aid in the management of the talon cusp. Small talon cusps are usually asymptomatic and need no treatment [16]. Large talon cusps may cause occlusal interference, irritation of the tongue during speech and mastication, displacement of the affected tooth, carious lesion in the developmental grooves delineating the cusp, pulpal necrosis, periapical pathosis, attrition of opposing tooth, and periodontal problems due to excessive occlusal forces [17]. The treatment of a talon cusp is dependent upon whether the cusp contains a pulp horn or not. However, a radiographic view is inherently difficult in tracing pulpal configuration inside the talon cusp because the cusp is superimposed over the affected tooth crown [6]. Hence, early diagnosis and sequential reduction of a talon cusp minimize the risk of pulp exposure thereby aiding in a secondary dentin formation.

## 4. Conclusion

Studies on prevalence of the talon cusp were mainly on the permanent dentition and very rare in the primary dentition. To avoid complications caused by the talon cusp, early diagnosis and treatment are recommended not only to improve aesthetics but also to restore the functional occlusion, prevent caries formation, and preserve the vitality of the affected tooth.
